# Recommendations for Evaluating Temporal Trends of Persistent Organic Pollutants in Breast Milk

**DOI:** 10.1289/ehp.1510219

**Published:** 2015-12-15

**Authors:** Tenzing Gyalpo, Martin Scheringer, Konrad Hungerbühler

**Affiliations:** 1 Safety and Environmental Technology Group, Swiss Federal Institute of Technology Zurich (ETH Zurich), Zurich, Switzerland; 2 Leuphana University of Lüneburg, Lüneburg, Germany

## Abstract

**Background:**

Biomonitoring data of persistent organic pollutants (POPs) in breast milk are increasingly collected and available for quantitative analysis of levels and time trends. A common approach is to apply log-linear regression to calculate doubling and halving times of the POP concentrations based on the temporal trend observed in breast milk. However, there are different, sometimes conflicting interpretations of these doubling and halving times.

**Objectives:**

We provide a mechanistic understanding of doubling and halving times where possible. Five recommendations are proposed for dealing with POP concentration trends in breast milk during three distinct periods (pre-ban, transition, post-ban period).

**Discussion:**

Using temporal trends of BDE-47 and PCB-153 in breast milk data, we show which information can be gained from the time-trend data. To this end, we analyzed time trends of hypothetical POPs for different periods with time-variant exposure and different intrinsic elimination half-lives, using a dynamic population-based pharmacokinetic model. Different pieces of information can be extracted from time-trend data from different periods. The analysis of trends of short-lived POPs is rather straightforward and facilitates extraction of the intrinsic elimination half-lives from the breast milk data. However, trends of slowly eliminated POPs only provide indications for the exposure time trend.

**Conclusions:**

Time-trend data of rapidly eliminated POPs provide information on exposure time trends and elimination half-lives. Temporal trends of slowly eliminated POPs are more complicated to interpret, and the extraction of exposure time trends and elimination half-lives require data sets covering several decades.

**Citation:**

Gyalpo T, Scheringer M, Hungerbühler K. 2016. Recommendations for evaluating temporal trends of persistent organic pollutants in breast milk. Environ Health Perspect 124:881–885; http://dx.doi.org/10.1289/ehp.1510219

## Introduction

The Stockholm Convention on Persistent Organic Pollutants (POPs) entered into force in 2004 and aims to protect humans and the environment from POPs (Stockholm Convention on Persistent Organic Pollutants 2009). To evaluate the effectiveness of measures taken under this Convention, time trends of POPs in human samples, mostly milk, are investigated. Today, many long-term data sets of POPs cover periods of 20–40 years; these include dichlorodiphenyltrichloroethane (DDT), polychlorinated biphenyls (PCBs), hexachlorobenzene (HCB), and polybrominated diphenyl ethers (PBDEs) (Fång et al. 2013; Glynn et al. 2012; Wilhelm et al. 2007).

**Figure 1:**
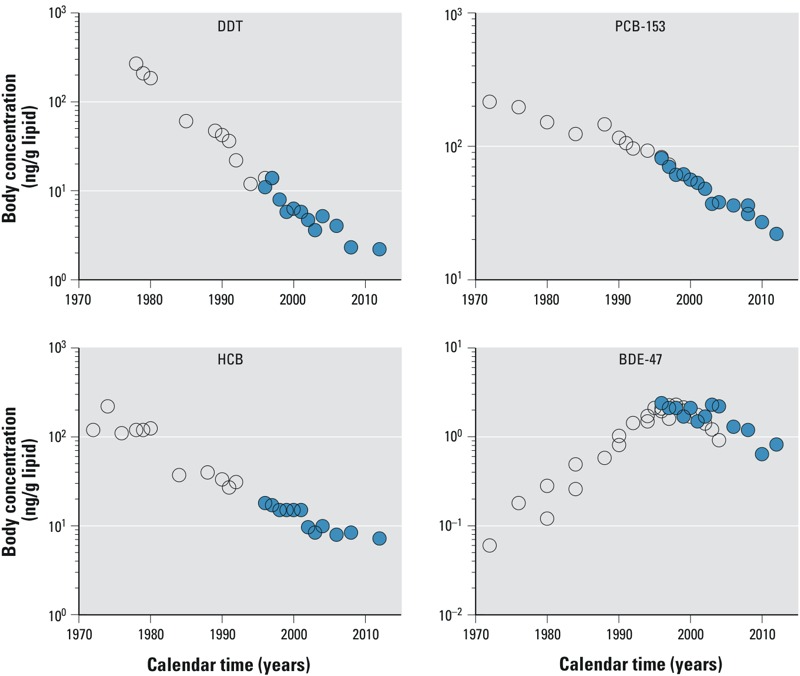
Selected POP concentrations in breast milk from Sweden sampled between 1972 and 2012 (open circles: Stockholm; closed circles: Uppsala) (Fängström et al. 2008; Glynn et al. 2012; Lignell et al. 2012, 2014; Meironyté et al. 1999; Norén and Meironyté 2000). Note the different scale on *y*-axis for BDE-47.

Here we call these time–concentration trends taken from groups of individuals with similar characteristics, but sampled in different years, “cross-sectional trend data” (CSTD). Declining CSTD are often fitted with exponential functions (Craan and Haines 1998; Glynn et al. 2012; Minh et al. 2004; Norén and Meironyté 2000). The slope of these fits provides the CSTD-based half-life, $t_{1/2}^{\mathrm{CSTD}}$. Generally, depending on the time period of data collection in relation to the introduction of the ban (or voluntary phaseout) of a chemical, and the physicochemical properties of the chemical and age of the population, the time–concentration plot may be subdivided into three different periods: pre-ban (constant positive slope), transition (gradual change in slope from positive to negative), and post-ban (constant negative slope). For example, the CSTD for BDE-47 in Figure 1 increase until the mid-1990s, when the phaseout of the technical mixture of pentaBDE was implemented in Sweden (Alcock and Busby 2006), then flattens out during the transition period, and eventually shows a negative slope during the post-ban period.

In the literature, different terms have been used to describe $t_{1/2}^{\mathrm{CSTD}}$, and various interpretations of $t_{1/2}^{\mathrm{CSTD}}$ have been proposed (Ritter et al. 2009). Technically, it is straightforward to derive $t_{1/2}^{\mathrm{CSTD}}$ from data of the post-ban period, but there is considerable confusion about the meaning of these CSTD-based half-lives. They were interpreted to be related to either the intrinsic elimination half-life, $t_{1/2}^{\mathrm{elim}}$, which indicates how fast the chemical is metabolized and excreted (elimination) from the human body (Noegrohati et al. 1992; Wolff et al. 2000), or to the trend in exposure characterized by the half-life of decline in intake, $t_{1/2}^{\mathrm{in}}$, which indicates how fast the total human exposure to the chemical is declining (e.g., time trend derived from total diet studies) (Glynn et al. 2012; Minh et al. 2004), or to both (Sjödin et al. 2004).

To resolve this confusion, Ritter et al. (2009) have provided a tool to disentangle these different half-lives. They developed a static population-based pharmacokinetic (PPK) model, called “CSTD half-life tool” (http://www.sust-chem.ethz.ch/downloads) specifically for the post-ban period that explains the relationships between $t_{1/2}^{\mathrm{in}}$, $t_{1/2}^{\mathrm{elim}}$, and $t_{1/2}^{\mathrm{CSTD}}$. “Static” here refers to the assumptions that there is no transfer of chemical from mother to child (i.e., *in utero* transfer or via breastfeeding), and that there is no change in body weight or lipid weight of any individual (Table 1, static PPK model). Because of these assumptions, the mass-balance equation of the model can be solved analytically (Ritter et al. 2009). This tool first derives $t_{1/2}^{\mathrm{CSTD}}$ from the exponential fit of a set of CSTD, but then in addition uses the relationships among $t_{1/2}^{\mathrm{in}}$, $t_{1/2}^{\mathrm{elim}}$, and $t_{1/2}^{\mathrm{CSTD}}$ to extract also $t_{1/2}^{\mathrm{elim}}$ from the data, which is another important metric for the assessment of human exposure to POP-like chemicals. This is a novel approach to estimating $t_{1/2}^{\mathrm{elim}}$ of a persistent chemical based on human data. However, limitations of the CSTD half-life tool due to the assumptions of the static PPK model were not specifically discussed in the original publication (Ritter et al. 2009) and will, therefore, be presented in this commentary.

**Table 1:** Comparison between static PPK and dynamic PPK models.

Processes	Static PPK model^*a*^	Dynamic PPK model^*b*^
*In utero* transfer	No	Yes
Transfer via breastfeeding	No	Yes
Change of body weight	No	Yes
Change of lipid weight	No	Yes
^***a***^The CSTD half-life tool as developed by Ritter et al. (2009) is one example of a static PPK model. The tool is available on http://www.sust-chem.ethz.ch/downloads. ^***b***^The PPK model as developed by Ritter et al. (2011) is one example of a dynamic PPK model.		

Meanwhile, dynamic PPK models that accommodate changes in individual characteristics with age and transgenerational transfer of chemicals (*in utero* exposure and via breastfeeding), such as the “CoZMoMAN model” or the “Ritter model,” have been developed and used to evaluate POP concentrations in longitudinal (Nøst et al. 2013) or cross-sectional biomonitoring data (Gyalpo et al. 2015; Ritter et al. 2011; Wong et al. 2013). CSTD collected under the Global Monitoring Plan of the Stockholm Convention (Secretariat of the Stockholm Convention 2016) can also be evaluated with these models, which (unlike the CSTD half-life tool) can accommodate transgenerational transfer and changes in body weight and lipid weight with age (Table 1, dynamic PPK model) and are not restricted to biomonitoring data from the post-ban period.

Here, our objective is to combine the knowledge gained from these previously published dynamic and static PPK models for the evaluation of CSTD. This is important because in the context of the Global Monitoring Plan of the Stockholm Convention (Secretariat of the Stockholm Convention 2016) extensive data sets have been collected and will be generated in the future, which calls for a common approach to interpreting the measured CSTD. To this end, we present five recommendations for the evaluation of CSTD sampled during the pre-ban and transition periods as observed, for example, for BDE-47 (Figure 1). In addition, we explain the limitations of the CSTD half-life tool and clarify its applicability domain, which is important for future applications of this tool. Hence, our overarching goal is to illustrate which model framework can be used in which situation to fully exploit the information that is contained in CSTD.

## Recommendations for the Evaluation of CSTD from Different Periods

We differentiate between two categories of POPs: *a*) POPs whose intrinsic elimination half-lives ($t_{1/2}^{\mathrm{elim}}$) are shorter than their intake doubling times ($t_{2}^{\mathrm{in}}$) and intake half-lives ($t_{1/2}^{\mathrm{in}}$) (e.g., BDE-47), and *b*) POPs whose $t_{1/2}^{\mathrm{elim}}$ values are longer than $t_{2}^{\mathrm{in}}$ and $t_{1/2}^{\mathrm{in}}$ (e.g., PCB-153). Thus, POPs similar to BDE-47 are referred to as “rapidly eliminated” or “short-lived” POPs, whereas POPs similar to PCB-153 are referred to as “slowly eliminated“ POPs. In the following sections we illustrate with the examples of BDE-47 and PCB-153 (Figure 1) and other POPs how the trends in CSTD from different periods are to be interpreted based on the insights gained from the dynamic PPK model. Five recommendations for the interpretation of CSTD sets are derived in the following sections. They are listed in Table 2.

**Table 2:** Recommendations for evaluation of CSTD.

Recommendations	Relevant time period
1. The doubling time in intake before the phaseout of the chemical can directly be derived from the slope of the exponential increase in CSTD, i.e., $t_{2}^{\mathrm{CSTD}}$ = $t_{2}^{\mathrm{in}}$, and is completely independent of $t_{1/2}^{\mathrm{elim}}$.	Pre-ban period
2. It does not make sense to estimate a $t_{1/2}^{\mathrm{CSTD}}$ during the transition period, even though it is technically possible.	Transition period
3. If there are indications that $t_{1/2}^{\mathrm{elim}}$ < $t_{1/2}^{\mathrm{in}}$, CSTD can be used to identify the half-life of decline in intake ($t_{1/2}^{\mathrm{CSTD}}$ = $t_{1/2}^{\mathrm{in}}$) already after 10 years into the transition period.	Transition period
4. The CSTD half-life tool is applicable not only to the post-ban period but also during the transition period if the chemical fulfills the condition of $t_{1/2}^{\mathrm{elim}}$ < $t_{1/2}^{\mathrm{in}}$, and CSTD are available for the later stage of the transition period.	Transition period
5. If there are indications for $t_{1/2}^{\mathrm{elim}}$ > $t_{1/2}^{\mathrm{in}}$ or long $t_{1/2}^{\mathrm{elim}}$ values in general (roughly ≥ 10 years), the CSTD half-life tool should not be applied.	Post-ban period

### Pre-ban Period

For newer POPs that were introduced to the market in the past 20 years, an exponential increase in CSTD is found in the population before the ban, for example, for PBDEs (Meironyté et al. 1999). Dynamic PPK models, as developed by Ritter et al. (2011) and also used by others (Wong et al. 2013), have shown that the doubling time of CSTD ($t_{2}^{\mathrm{CSTD}}$) directly reflects the doubling time of the intake ($t_{2}^{\mathrm{in}}$), that is, $t_{2}^{\mathrm{CSTD}}$ = $t_{2}^{\mathrm{in}}$. Importantly, the value of $t_{2}^{\mathrm{CSTD}}$ = $t_{2}^{\mathrm{in}}$ is not affected by the intrinsic elimination half-life, $t_{1/2}^{\mathrm{elim}}$. That is, if intake estimates of BDE-47 before 1995 had been reported for the Swedish population, for example from total diet studies, they would have increased with the same slope as the CSTD measured in the pre-ban period (Figure 1).

Sampling from breast milk is restricted to lactating women of a certain age (mostly 20–40 years). CSTD from blood samples are, however, equally valid and appropriate for elucidating time trends. For instance, the pre-ban CSTD of serum samples of 40- to 50-year-old Norwegian men provide a good estimate of the doubling time of PBDE intake by the Norwegian population (Thomsen et al. 2002). Thus, our first recommendation is: **The doubling time in intake before the phaseout of the chemical can directly be derived from the slope of the exponential increase in CSTD, that is, $t_{2}^{\mathrm{CSTD}}$ = $t_{2}^{\mathrm{in}}$, and is completely independent of $t_{1/2}^{\mathrm{elim}}$.** That is, in the pre-ban period, all individuals of a population experience the same doubling time of their exposure versus calendar time. Note that the absolute intake rate (e.g., in nanograms per kilogram per day) is age-dependent.

### Transition Period

**Figure 2:**
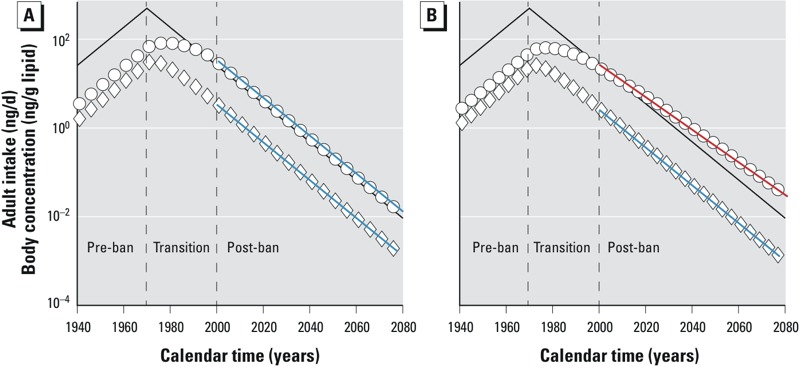
Modeled CSTD of two hypothetical chemicals in 30-year-old individuals with identical intake trend (black line, $t_{2}^{\mathrm{in}}$ = $t_{1/2}^{\mathrm{in}}$ = 7 years) for the period 1940–2080. Circles: slow elimination, $t_{1/2}^{\mathrm{elim}}$ = 14 years; diamonds: rapid elimination, $t_{1/2}^{\mathrm{elim}}$ = 3 years. Ban of chemicals took place in 1970. (*A*) If the static PPK model is applied, the slopes of the CSTD of both chemicals (slopes indicated by blue lines) are parallel to the intake trend in the post-ban period. (*B*) If the dynamic PPK model is applied, only the slope of the CSTD of the rapidly eliminated chemical (slope indicated by blue line) is parallel to the intake trend in the post-ban period. The slope of the CSTD of the slowly eliminated chemical (slope indicated by red line) deviates from the others.

In this period, calculation of a $t_{1/2}^{\mathrm{CSTD}}$ always results in a very long $t_{1/2}^{\mathrm{CSTD}}$. For instance, $t_{1/2}^{\mathrm{CSTD}}$ for BDE-47 is 26.7 years for the period of 1996–2003 (Lignell et al. 2014) or 16.5 years for 1996–2006 (Lignell et al. 2009). In both cases $t_{1/2}^{\mathrm{CSTD}}$ was calculated for the first 10 years of the transition period, when concentrations are rather stable. Similarly, the CSTD of hexabromocyclododecane (HBCDD) from Swedish mothers can also be allocated to the end of the pre-ban and the beginning of the transition period (Covaci et al. 2006; Fängström et al. 2005). Consequently, very long $t_{1/2}^{\mathrm{CSTD}}$ values (i.e., 15–27.7 years) were estimated for 1996–2010 and 2002–2012, respectively (Lignell et al. 2012, 2014). Hence, our second recommendation is: **It does not make sense to estimate $t_{1/2}^{\mathrm{CSTD}}$ during the transition period, even though it is technically possible.** For rapidly eliminated chemicals this restriction applies only to the beginning of the transition period (see below), but for slowly eliminated chemicals the derivation of $t_{1/2}^{\mathrm{CSTD}}$ should be avoided for the whole transition period. The longer $t_{1/2}^{\mathrm{elim}}$ is, the slower is the change from increasing to decreasing CSTD during the transition period (Figure 2).

However, for rapidly eliminated chemicals, it is possible to calculate a meaningful value of $t_{1/2}^{\mathrm{CSTD}}$ already at the end of the transition period because $t_{1/2}^{\mathrm{CSTD}}$ is then already equal to $t_{1/2}^{\mathrm{in}}$ for these chemicals. For example, after approximately 10 years into the transition period, the $t_{1/2}^{\mathrm{CSTD}}$ of BDE-47 reduces to 6.4 years for the period of 2004–2012 (see Table S1 for empirical CSTD and fitted $t_{1/2}^{\mathrm{CSTD}}$). Estimates for $t_{1/2}^{\mathrm{in}}$ from Swedish food baskets reveal a $t_{1/2}^{\mathrm{in}}$ value of 6.8 years for the period of 1999–2010 (Darnerud et al. 2006; National Food Agency 2012; Törnkvist et al. 2011), which is very close to the 6.4 years found for $t_{1/2}^{\mathrm{CSTD}}$. The reason why we find this result already around 10 years into the transition period is that, for BDE-47, $t_{1/2}^{\mathrm{elim}}$ < $t_{1/2}^{\mathrm{in}}$: estimates of $t_{1/2}^{\mathrm{elim}}$ of BDE-47 are rather short, between 1.4 and 3.0 years (Geyer et al. 2004; Trudel et al. 2011), and clearly shorter than the $t_{1/2}^{\mathrm{in}}$ of 6.4 years. Thus, our third recommendation is: **If there are indications that $t_{1/2}^{\mathrm{elim}}$ < $t_{1/2}^{\mathrm{in}}$, CSTD can be used to identify the half-life of decline in intake ($t_{1/2}^{\mathrm{CSTD}}$ = $t_{1/2}^{\mathrm{in}}$) already after 10 years into the transition period.** Ritter et al. (2009) stated that if only CSTD from the post-ban period are considered, $t_{1/2}^{\mathrm{CSTD}}$ is equal to $t_{1/2}^{\mathrm{in}}$. For chemicals like BDE-47, this is true already after around 10 years into the transition period.

If we now apply the CSTD half-life tool to derive $t_{1/2}^{\mathrm{elim}}$ from the CSTD of BDE-47 from the period of 2004–2012, we obtain a $t_{1/2}^{\mathrm{elim}}$ value of 2.2 years for BDE-47 (see Table S1 for input data used and model output), which agrees very well with estimates from previous studies—specifically, 1.4 and 3.0 years from Geyer et al. (2004) and Trudel et al. (2011), respectively. Consequently, our fourth recommendation is: **The CSTD half-life tool is applicable not only to the post-ban period but also during the transition period if the chemical fulfills the condition of $t_{1/2}^{\mathrm{elim}}$ < $t_{1/2}^{\mathrm{in}}$, and CSTD are available for the later stage of the transition period.**

The CSTD of DDT from studies of Swedish mothers (Glynn et al. 2012; Lignell et al. 2014; Norén and Meironyté 2000) (see Table S2) illustrate our fourth recommendation. Based on CSTD of DDT from the post-ban period (1996–2006), the CSTD half-life tool estimates a $t_{1/2}^{\mathrm{elim}}$ of 2.2 years (Ritter et al. 2009). When we apply the half-life tool to CSTD from the later stage of the transition period (1980–2006, leaving out the first decade of the transition period from 1970 to 1980), we obtain a $t_{1/2}^{\mathrm{elim}}$ of 1.9 years (see Table S2 for input data used and model output), which is very close to the estimate of 2.2 years derived from the post-ban data.

The same will probably apply to HBCDD in the near future. Efforts to reduce HBCDD emissions to the environment were initiated around 2004 in Sweden (Remberger et al. 2004). Estimates of $t_{1/2}^{\mathrm{elim}}$ of HBCDD in humans are only a few months (Geyer et al. 2004), which is most likely shorter than $t_{1/2}^{\mathrm{in}}$ of HBCDD. Therefore, as soon as HBCDD intake decreases due to reductions in emission, the CSTD half-life tool will be suitable for estimating $t_{1/2}^{\mathrm{elim}}$ based on future CSTD of HBCDD from the general population.

### Post-ban Period

Ritter et al. (2009) demonstrated by using a static PPK model that in the post-ban period $t_{1/2}^{\mathrm{CSTD}}$ = $t_{1/2}^{\mathrm{in}}$ is valid. Under the assumption of “static” individuals—no chemical transfer via *in utero* exposure or via breastfeeding and no change in body weight and lipid weight—this result is true without any qualifications. However, as soon as there is transfer of chemical from mother to child, this result is true only if $t_{1/2}^{\mathrm{elim}}$ < $t_{1/2}^{\mathrm{in}}$. If this condition is not fulfilled because $t_{1/2}^{\mathrm{elim}}$ is very long, the measured CSTD violate the assumptions of the CSTD half-life tool, and estimates derived with this tool will be incorrect. For example, when CSTD for PCB-153 (see Table S3) are inputted into the CSTD half-life tool, the estimated $t_{1/2}^{\mathrm{CSTD}}$ value is 9.8 years, and the estimated $t_{1/2}^{\mathrm{elim}}$ is 7.0 years. This value of $t_{1/2}^{\mathrm{elim}}$ for PCB-153 is considerably shorter than previous estimates of 14.4–17 years (Aylward et al. 2014; Bu et al. 2015; Ritter et al. 2011), and thus appears to be incorrect. Additionally, PCB-153 concentrations during the post-ban period (25–30 years after the highest concentrations had occurred, i.e., since around 1995) have been reported to increase with age within cross-sectional populations (Quinn and Wania 2012; Ritter et al. 2011), which is possible only if $t_{1/2}^{\mathrm{elim}}$ > $t_{1/2}^{\mathrm{in}}$ (Ritter et al. 2011). Therefore, our fifth recommendation is: **If there are indications that $t_{1/2}^{\mathrm{elim}}$ > $t_{1/2}^{\mathrm{in}}$ or long $t_{1/2}^{\mathrm{elim}}$ values in general (roughly ≥ 10 years), the CSTD half-life tool should not be applied.**

The reason why the CSTD half-life tool is not applicable to PCB-153 and other chemicals with long $t_{1/2}^{\mathrm{elim}}$ is that, due to the long $t_{1/2}^{\mathrm{elim}}$ of the chemical, the body burden later in life is still influenced by the exposure to the chemical much earlier in life (i.e., from *in utero* exposure and transfer via breastfeeding). This fact is not considered in the assumptions made in the CSTD half-life tool (Table 1, static PPK model). A more realistic model is a dynamic PPK model. Such a model is not restricted to the post-ban period but includes the pre-ban and transition periods, and longitudinal POP concentrations are estimated for each individual, including transgenerational transfer of chemical from mother to child (Table 1, dynamic PPK model). Figure 2 compares modeled CSTD with the assumptions of the CSTD half-life tool (*A*) and under more realistic assumptions (*B*) for two hypothetical chemicals. Importantly, as illustrated in Figure 2B, for chemicals whose $t_{1/2}^{\mathrm{elim}}$ exceeds $t_{1/2}^{\mathrm{in}}$ (circles), the slope in CSTD is not equal to the slope in intake of the chemical at any time in the post-ban period: that is $t_{1/2}^{\mathrm{CSTD}}$ ≠ $t_{1/2}^{\mathrm{in}}$. In contrast, for chemicals with $t_{1/2}^{\mathrm{elim}}$ ≤ $t_{1/2}^{\mathrm{in}}$, $t_{1/2}^{\mathrm{CSTD}}$ = $t_{1/2}^{\mathrm{in}}$ is true (diamonds). This shift in the slope for slowly eliminated chemicals in Figure 2B (red line) is due to the non-zero initial concentration at birth and intake via breastfeeding. The effect of transgenerational input is pronounced in the post-ban period, when intake is declining and therefore the contribution from a “contaminated” mother is important.

Another case that illustrates the limitations of the CSTD half-life tool is HCB. Two studies have reported a $t_{1/2}^{\mathrm{elim}}$ of HCB of around 6 years (Bu et al. 2015; To-Figueras et al. 2000), and the $t_{1/2}^{\mathrm{in}}$ is 12.0 years for the period of 1975–2010 in Sweden (Darnerud et al. 2006; National Food Agency 2012; Törnkvist et al. 2011; Vaz 1995). When the CSTD and the intake data from Table S4 are inputted, the CSTD half-life tool estimates a $t_{1/2}^{\mathrm{CSTD}}$ value of 14.9 years, and a $t_{1/2}^{\mathrm{elim}}$ value of only 2.4 years, which is considerably shorter than previous $t_{1/2}^{\mathrm{elim}}$ estimates of approximately 6 years (Bu et al. 2015; To-Figueras et al. 2000). As for PCB-153, cross-sectional age–concentration trends should be evaluated to confirm the model outputs of the CSTD half-life tool. However, this cross-check can be performed only with cross-sectional data from the post-ban period, because age–concentration trends will not differ between slowly and rapidly eliminated POPs during the pre-ban and transition periods (Gyalpo et al. 2015; Quinn and Wania 2012). If $t_{1/2}^{\mathrm{elim}}$ is substantially shorter than $t_{1/2}^{\mathrm{in}}$, as suggested by the CSTD half-life tool estimates for HCB, HCB concentrations should not increase with age in cross-sectional populations. However, cross-sectional biomonitoring data from Australia (Bu et al. 2015), Spain (Zubero et al. 2015), and Germany (Becker et al. 2002) do show increasing HCB concentration with increasing age, indicating that $t_{1/2}^{\mathrm{elim}}$ is underestimated by the CSTD half-life tool. Hence, it is advisable not to use this tool for evaluating CSTD of HCB.

## Conclusions

In evaluating decreasing CSTD, it is important to distinguish between three half-lives: the CSTD-based half-life ($t_{1/2}^{\mathrm{CSTD}}$), the half-life of decline in intake ($t_{1/2}^{\mathrm{in}}$), and the intrinsic elimination half-life ($t_{1/2}^{\mathrm{elim}}$). During the pre-ban period, the doubling time of CSTD (*t*_2_^CSTD^) is equal to the doubling time of intake ($t_{2}^{\mathrm{in}}$); during the transition period, calculation of $t_{1/2}^{\mathrm{CSTD}}$ yields nonsensical results; and in the post-ban period, $t_{1/2}^{\mathrm{CSTD}}$ is equal to $t_{1/2}^{\mathrm{in}}$ only for chemicals that are rapidly eliminated, whereas for slowly eliminated chemicals, $t_{1/2}^{\mathrm{CSTD}}$ only represents the upper limit of $t_{1/2}^{\mathrm{in}}$. Importantly, $t_{1/2}^{\mathrm{CSTD}}$ never equals $t_{1/2}^{\mathrm{elim}}$.

For chemicals for which estimates of short $t_{1/2}^{\mathrm{elim}}$ exist (e.g., extrapolated from animal studies or derived from highly exposed individuals), the CSTD half-life tool will provide a good estimate of $t_{1/2}^{\mathrm{elim}}$ based on CSTD from the later stage of the transition period. In contrast, for chemicals that may have long $t_{1/2}^{\mathrm{elim}}$ values, $t_{1/2}^{\mathrm{elim}}$ can be derived only with dynamic PPK models combined with sequential sets of cross-sectional data. This approach requires long-term planning since cross-sectional data sets are needed from at least 20 years after the ban of the chemical.

As pointed out by Ritter et al. (2009), the $t_{1/2}^{\mathrm{CSTD}}$ is specific to the sampled population. Different countries can have different $t_{1/2}^{\mathrm{CSTD}}$ values for the same chemicals because $t_{1/2}^{\mathrm{CSTD}}$ is a measure of the degree of the reduction in exposure to a chemical, which is governed by the country’s amount in production and use and the time of a phaseout. It is an “apparent” property that is specific to the environmental conditions in the country and therefore not something that has to be globally identical.

## Supplemental Material

(256 KB) PDF

## References

[r1] Alcock RE, Busby J (2006). Risk migration and scientific advance: the case of flame-retardant compounds.. Risk Anal.

[r2] Aylward LL, Collins JJ, Bodner KM, Wilken M, Bodnar CM (2014). “Intrinsic” elimination rate and dietary intake estimates for selected indicator PCBs: toxicokinetic modeling using serial sampling data in US subjects, 2005–2010.. Chemosphere.

[r3] Becker K, Kaus S, Krause C, Lepom P, Schulz C, Seiwert M (2002). German Environmental Survey 1998 (GerES III): environmental pollutants in blood of the German population.. Int J Hyg Envrion Health.

[r4] Bu Q, MacLeod M, Wong F, Toms LML, Mueller JF, Yu G (2015). Historical intake and elimination of polychlorinated biphenyls and organochlorine pesticides by the Australian population reconstructed from biomonitoring data.. Environ Int.

[r5] Covaci A, Gerecke AC, Law RJ, Voorspoels S, Kohler M, Heeb NV (2006). Hexabromocyclododecanes (HBCDs) in the environment and humans: a review.. Environ Sci Technol.

[r6] Craan AG, Haines DA (1998). Twenty-five years of surveillance for contaminants in human breast milk.. Arch Environ Contam Toxicol.

[r7] Darnerud PO, Atuma S, Aune M, Bjerselius R, Glynn A, Grawé KP (2006). Dietary intake estimations of organohalogen contaminants (dioxins, PCB, PBDE and chlorinated pesticides, e.g. DDT) based on Swedish market basket data.. Food Chem Toxicol.

[r8] Fång J, Nyberg E, Bignert A, Bergman Å (2013). Temporal trends of polychlorinated dibenzo-*p*-dioxins and dibenzofurans and dioxin-like polychlorinated biphenyls in mothers’ milk from Sweden, 1972–2011.. Environ Int.

[r9] Fängström B, Athanassiadis I, Odsjö T, Norén K, Bergman Å (2008). Temporal trends of polybrominated diphenyl ethers and hexabromocyclododecane in milk from Stockholm mothers, 1980–2004.. Mol Nutr Food Res.

[r10] Fängström B, Hovander L, Bignert A, Athanassiadis I, Linderholm L, Grandjean P (2005). Concentrations of polybrominated diphenyl ethers, polychlorinated biphenyls, and polychlorobiphenylols in serum from pregnant Faroese women and their children 7 years later.. Environ Sci Technol.

[r11] Geyer HJ, Schramm KW, Darnerud PO, Aune M, Feicht EA, Fried KW (2004). Terminal elimination half-lives of the brominated flame retardants TBBPA, HBCD, and lower brominated PBDEs in humans.. Organohalogen Compounds.

[r12] Glynn A, Lignell S, Aune M, Darnerud PO, Törnkvist A (2012). Temporal trends of organohalogen compounds in mother’s milk from Sweden.. In: Global Contamination Trends of Persistent Organic Chemicals (Loganathan BG, Lam PKS, eds).

[r13] Gyalpo T Toms LML Mueller JF Harden FA Scheringer M Hungerbühler K 2015 Insights into PBDE uptake, body burden, and elimination gained from Australian age–concentration trends observed shortly after peak exposure. Environ Health Perspect 123 978 984, 10.1289/ehp.1408960 25768049 PMC4590757

[r14] Lignell S, Aune M, Darnerud PO, Cnattingius S, Glynn A (2009). Persistent organochlorine and organobromine compounds in mother’s milk from Sweden 1996–2006: compound-specific temporal trends.. Environ Res.

[r15] Lignell S, Aune M, Glynn A, Cantillana T, Fridén U (2012). Levels of Persistent Halogenated Organic Pollutants (POP) in Mother’s Milk from First-Time Mothers in Uppsala, Sweden – Results from 2008/2010 and Temporal Trends 1996–2010.. http://www.diva-portal.org/smash/get/diva2:710423/FULLTEXT01.pdf.

[r16] Lignell S, Aune M, Glynn A, Cantillana T, Fridén U (2014). Levels of Persistent Halogenated Organic Pollutants (POP) in Mother’s Milk from First-Time Mothers in Uppsala, Sweden: Results from Year 2012 and Temporal Trends for the Time Period 1996–2012.. http://www.imm.ki.se/Datavard/Rapporter/Sakrapport_trend9612.pdf.

[r17] Meironyté D, Norén K, Bergman A (1999). Analysis of polybrominated diphenyl ethers in Swedish human milk. A time-related trend study, 1972–1997.. J Toxicol Environ Health A.

[r18] Minh NH, Someya M, Minh TB, Kunisue T, Iwata H, Watanabe M (2004). Persistent organochlorine residues in human breast milk from Hanoi and Hochiminh city, Vietnam: contamination, accumulation kinetics and risk assessment for infants.. Environ Pollut.

[r19] National Food Agency (2012). Market Basket 2010 – Chemical Analysis, Exposure Estimation and Health-Related Assessment of Nutrients and Toxic Compounds in Swedish Food Baskets.. http://www.livsmedelsverket.se/globalassets/rapporter/2010/2012_livsmedelsverket_7_market_basket_2010.pdf.

[r20] Noegrohati S, Sardjoko, Untung K, Hammers WE. 1992 Impact of DDT spraying on the residue levels in soil, chicken, fishpond water, carp and human-milk samples from malaria infested villages in Central Java. Toxicol Environ Chem 34 237 251

[r21] Norén K, Meironyté D (2000). Certain organochlorine and organobromine contaminants in Swedish human milk in perspective of past 20–30 years.. Chemosphere.

[r22] Nøst TH Breivik K Fuskevåg OM Nieboer E Odland JØ Sandanger TM 2013 Persistent organic pollutants in Norwegian men from 1979 to 2007: intraindividual changes, age–period–cohort effects, and model predictions. Environ Health Perspect 121 1292 1298, doi:10.1289/ehp.1206317 24007675 PMC3855502

[r23] Quinn CL Wania F 2012 Understanding differences in the body burden–age relationships of bioaccumulating contaminants based on population cross sections versus individuals. Environ Health Perspect 120 554 559, doi:10.1289/ehp.1104236 22472302 PMC3339463

[r24] Remberger M, Sternbeck J, Palm A, Kaj L, Strömberg K, Brorström-Lundén E (2004). The environmental occurrence of hexabromocyclododecane in Sweden.. Chemosphere.

[r25] Ritter R Scheringer M MacLeod M Moeckel C Jones KC Hungerbühler K 2011 Intrinsic human elimination half-lives of polychlorinated biphenyls derived from the temporal evolution of cross-sectional biomonitoring data from the United Kingdom. Environ Health Perspect 119 225 231, doi:10.1289/ehp.1002211 20934951 PMC3040610

[r26] Ritter R Scheringer M MacLeod M Schenker U Hungerbühler K 2009 A multi-individual pharmacokinetic model framework for interpreting time trends of persistent chemicals in human populations: application to a postban situation. Environ Health Perspect 117 1280 1286, doi:10.1289/ehp.0900648 19672409 PMC2721873

[r27] Secretariat of the Stockholm Convention (2016). http://chm.pops.int/Implementation/GlobalMonitoringPlan/Overview/tabid/83/Default.aspx.

[r28] Sjödin A Jones RS Focant JF Lapeza C Wang RY McGahee EE 2004 Retrospective time-trend study of polybrominated diphenyl ether and polybrominated and polychlorinated biphenyl levels in human serum from the United States. Environ Health Perspect 112 654 658, doi:10.1289/ehp.6826 15121506 PMC1241957

[r29] Stockholm Convention on Persistent Organic Pollutants (2009). Stockholm Convention on Persistent Organic Pollutants (POPs), as amended in 2009.. http://www.env.go.jp/chemi/pops/treaty/treaty_en2009.pdf.

[r30] Thomsen C, Lundanes E, Becher G (2002). Brominated flame retardants in archived serum samples from Norway: a study on temporal trends and the role of age.. Environ Sci Technol.

[r31] To-Figueras J, Barrot C, Sala M, Otero R, Silva M, Ozalla MD (2000). Excretion of hexachlorobenzene and metabolites in feces in a highly exposed human population.. Environ Health Perspect.

[r32] Törnkvist A, Glynn A, Aune M, Darnerud PO, Ankarberg EH (2011). PCDD/F, PCB, PBDE, HBCD and chlorinated pesticides in a Swedish market basket from 2005 – levels and dietary intake estimations.. Chemosphere.

[r33] Trudel D, Scheringer M, von Goetz N, Hungerbühler K (2011). Total consumer exposure to polybrominated diphenyl ethers in North America and Europe.. Environ Sci Technol.

[r34] Vaz R (1995). Average Swedish dietary intakes of organochlorine contaminants via foods of animal origin and their relation to levels in human milk, 1975–90.. Food Addit Contam.

[r35] Wilhelm M, Ewers U, Wittsiepe J, Fürst P, Hölzer J, Eberwein G (2007). Human biomonitoring studies in North Rhine-Westphalia, Germany.. Int J Hyg Envrion Health.

[r36] Wolff MS, Zeleniuch-Jacquotte A, Dubin N, Toniolo P (2000). Risk of breast cancer and organochlorine exposure.. Cancer Epidemiol Biomarkers Prev.

[r37] Wong F, Cousins IT, MacLeod M (2013). Bounding uncertainties in intrinsic human elimination half-lives and intake of polybrominated diphenyl ethers in the North American population.. Environ Int.

[r38] Zubero MB, Aurrekoetxea JJ, Murcia M, Ibarluzea JM, Goñi F, Jiménez C (2015). Time trends in serum organochlorine pesticides and polychlorinated biphenyls in the general population of Biscay, Spain.. Arch Environ Contam Toxicol.

