# Supplementary material for: Recommendations for Evaluating Temporal Trends of Persistent Organic Pollutants in Breast Milk
Source: Environ Health Perspect. 2015 Dec 15;124(7):881–5. doi: 10.1289/ehp.1510219 (PMC4937868; doi:10.1289/ehp.1510219)
Supplement: (256 KB) PDF [file ehp.1510219.s001.acco.pdf]

Environ Health Perspect
DOI: 10.1289/ehp.1510219

Note to readers with disabilities: *EHP* strives to ensure that all journal content is accessible to all readers. However, some figures and Supplemental Material published in *EHP* articles may not conform to 508 standards due to the complexity of the information being presented. If you need assistance accessing journal content, please contact ehp508@niehs.nih.gov. Our staff will work with you to assess and meet your accessibility needs within 3 working days.

Supplemental Material

Recommendations for Evaluating Temporal Trends of Persistent Organic Pollutants in Breast Milk

Tenzing Gyalpo, Martin Scheringer, and Konrad Hungerbühler

Table of Contents

Table S1: Input data for and model output of CSTD half-life tool for BDE-47 (tool available on http://www.sust-chem.ethz.ch/downloads)
Table S2: Input data for and model output of CSTD half-life tool for DDT (tool available on http://www.sust-chem.ethz.ch/downloads)
Table S3: Input data for and model output of CSTD half-life tool for PCB-153 (tool available on http://www.sust-chem.ethz.ch/downloads)
Table S4: Input data for and model output of CSTD half-life tool for HCB (tool available on http://www.sust-chem.ethz.ch/downloads)
References

Table S1: Input data for and model output of the CSTD half-life tool for BDE-47 (tool available on http://www.sust-chem.ethz.ch/downloads).

**Input data**	Year	Concentration (ng/g lipid)	Reference
	2004	1.7[a]	Glynn et al. (2012)/Fangström et al. (2008)
	2006	1.3	Glynn et al. (2012)
	2008	1.2	Lignell et al. (2012)
	2010	0.64	Lignell et al. (2012)
	2012	0.82	Lignell et al. (2014)
**Input data**	Year	Intake (ng/d)	Reference
	1999	26.5	Darnerud et al. (2006)
	2005	20.6	Törnkvist et al. (2011)
	2010	8.46	National Food Agency (2012)
**Model output**	CSTD-based half-life		
	$t_{1/2}^{\mathrm{CSTD}}$	6.4 years	
	Intrinsic elimination half-life		
	$t_{1/2}^{\mathrm{elim}}$	2.2 years	

[a]weighted average

Table S2: Input data for and model output of CSTD half-life tool for DDT (tool available on http://www.sust-chem.ethz.ch/downloads).

**Input data**	Year	Concentration (ng/g lipid)	Reference
	1980	185	Norén and Meironyté (2000)
	1985	61	Norén and Meironyté (2000)
	1989	47	Norén and Meironyté (2000)
	1990	42	Norén and Meironyté (2000)
	1991	36	Norén and Meironyté (2000)
	1992	22	Norén and Meironyté (2000)
	1994	12	Norén and Meironyté (2000)
	1996	12.5[a]	Glynn et al. (2012)/Norén and Meironyté (2000)
	1997	14[a]	Glynn et al. (2012)/(Norén and Meironyté (2000)
	1998	7.9	Glynn et al. (2012)
	1999	5.8	Glynn et al. (2012)
	2000	6.3	Glynn et al. (2012)
	2001	5.8	Glynn et al. (2012)
	2002	4.7	Glynn et al. (2012)
	2003	3.6	Glynn et al. (2012)
	2004	5.2	Glynn et al. (2012)
	2006	4	Glynn et al. (2012)
	2008	2.3	Glynn et al. (2012)
	2012	2.2	Lignell et al. (2014)
**Input data**	Year	Intake (ng/d)	Reference
	1999	76.5	Darnerud et al. (2006) (25% of *p,p'*-DDE intake)[b]
	2005	52.5	Törnkvist et al. (2011) (25% of *p,p'*-DDE intake)[b]
**Model output**	CSTD-based half-life		
	$t_{1/2}^{\mathrm{CSTD}}$	4.7 years	
	Intrinsic elimination half-life		
	$t_{1/2}^{\mathrm{elim}}$	1.9 years	

[a]weighted average
[b]same approach as in Ritter et al. (2009)

Table S3: Input data for and model output of CSTD half-life tool for PCB-153 (tool available on http://www.sust-chem.ethz.ch/downloads).

**Input data**	Year	Concentration (ng/g lipid)	Reference
	2000	56	Glynn et al. (2012)
	2001	53	Glynn et al. (2012)
	2002	48	Glynn et al. (2012)
	2003	37	Glynn et al. (2012)
	2004	38	Glynn et al. (2012)
	2006	36	Glynn et al. (2012)
	2008	33.5[a]	Glynn et al. (2012)/Lignell et al. (2012)
	2010	27	Lignell et al. (2012)
	2012	22	Lignell et al. (2014)
**Input data**	Year	Intake (ng/d)	Reference
	1999	139	Darnerud et al. 2006
	2005	85.3	Törnkvist et al. 2011
	2010	89.3	National Food Agency (2012)
**Model output**	CSTD-based half-life		
	$t_{1/2}^{\mathrm{CSTD}}$	9.8 years	
	Intrinsic elimination half-life		
	$t_{1/2}^{\mathrm{elim}}$	7.0 years	

[a]weighted average

Table S4: Input data for and model output of CSTD half-life tool for HCB (tool available on http://www.sust-chem.ethz.ch/downloads).

**Input data**	Year	Concentration (ng/g lipid)	Reference
	2001	15	Glynn et al. (2012)
	2002	9.6	Glynn et al. (2012)
	2003	8.4	Glynn et al. (2012)
	2004	10	Glynn et al. (2012)
	2006	7.9	Glynn et al. (2012)
	2008	8.4	Glynn et al. (2012)
	2012	7.2	Lignell et al. (2014)
**Input data**	Year	Intake (ng/d)	Reference
	1975	720	Vaz et al. (1995)[a]
	1980	480	Vaz et al. (1995)
	1985	180	Vaz et al. (1995)
	1990	300	Vaz et al. (1995)
	1994	330	Darnerud et al. (2006)
	1999	114	Darnerud et al. (2006)
	2005	83.4	Törnkvist et al. (2011)
	2010	97.7	National Food Agency (2012)
**Model output**	CSTD-based half-life		
	$t_{1/2}^{\mathrm{CSTD}}$	14.9 years	
	Intrinsic elimination half-life		
	$t_{1/2}^{\mathrm{elim}}$	2.4 years	

[a]Vaz et al. (1995) expressed intakes as ng/kg/d assuming a body weight of 60 kg.

References

Darnerud PO, Atuma S, Aune M, Bjerselius R, Glynn A, Grawe KP, et al. 2006. Dietary intake estimations of organohalogen contaminants (dioxins, PCB, PBDE and chlorinated pesticides, e.g. DDT) based on Swedish market basket data. Food Chem Toxicol 44:1597–1606.

Fängström B, Athanassiadis I, Odsjö T, Norén K, Bergman Å. 2008. Temporal trends of polybrominated diphenyl ethers and hexabromocyclododecane in milk from Stockholm mothers, 1980–2004. Mol Nutr Food Res 52:187–193.

Glynn A, Lignell S, Darnerud PO, Törnkvist A. 2012. Temporal trends of organohalogen compounds in mother's milk from Sweden. In: Global gontamination trends of persistent organic chemicals, (Loganathan BG, Lam PKS, eds). Boca Raton:CRC Press.

Lignell S, Aune M, Glynn A, Cantillana T, Fridén U. 2012. Levels of persistent halogenated organic pollutants (POP) in mother's milk from first-time mothers in Uppsala, Sweden – Results from 2008/2010 and temporal trends 1996–2010. Uppsala:Swedish Environmental Protection Agency.

Lignell S, Aune M, Glynn A, Cantillana T, Fridén U. 2014. Levels of persistent halogenated organic pollutants (POP) in mother's milk from first-time mothers in Uppsala, Sweden: results from year 2012 and temporal trends for the time period 1996–2012. Uppsala:Swedish Environmental Protection Agency.

National Food Agency. 2012. Market Basket 2010 – Chemical analysis, exposure estimation and health-related assessment of nutrients and toxic compounds in Swedish food baskets. Uppsala:National Food Agency.

Norén K, Meironyté D. 2000. Certain organochlorine and organobromine contaminants in Swedish breast milk in perspective of past 20–30 years. Chemosphere 40:1111–1123.

Ritter R, Scheringer M, MacLeod M, Schenker U, Hungerbühler K. 2009. A multi-individual pharmacokinetic model framework for interpreting time trends of persistent chemicals in human populations: Application to a postban situation. Environ Health Perspect 117:1280–1286.

Törnkvist A, Glynn A, Aune M, Darnerud PO, Ankarberg EH. 2011. PCDD/F, PCB, PBDE, HBCD and chlorinated pesticides in a Swedish market basket from 2005 – Levels and dietary intake estimations. Chemosphere 83:193–199.

Vaz R. 1995. Average Swedish dietary intakes of organochlorine contaminants via foods of animal origin and their relation to levels in breast milk, 1975–90. Food Addit Contam 12:543–558.
